# Cepharanthine Ameliorates Chondrocytic Inflammation and Osteoarthritis *via* Regulating the MAPK/NF-κB-Autophagy Pathway

**DOI:** 10.3389/fphar.2022.854239

**Published:** 2022-06-21

**Authors:** Minjun Yao, Caihua Zhang, Lingzhi Ni, Xiaoxiao Ji, Jianqiao Hong, Yazhou Chen, Jie Wang, Congsun Li, Jiyan Lin, Tingting Lu, Yihao Sheng, Menghao Sun, Mingmin Shi, Chenhe Zhou, Xunzi Cai

**Affiliations:** ^1^ Department of Orthopedic Surgery, Second Affiliated Hospital, School of Medicine, Zhejiang University, Hangzhou, China; ^2^ Orthopedic Research Institute of Zhejiang University, Hangzhou, China; ^3^ Department of Orthopedics, Ningbo First Hospital, Ningbo, China; ^4^ Department of Orthopedics, Hangzhou Third Hospital, Hangzhou, China; ^5^ Department of Oncology, The First Clinical Medical College of Wenzhou Medical University, Wenzhou, China; ^6^ Department of Orthopedics, Hangzhou Xiaoshan Cha Ting Orthopedic Trauma Hospital, Hangzhou, China

**Keywords:** cepharanthine, chondrocytes, osteoarthritis, MAPK, NF-κB, autophagy

## Abstract

Osteoarthritis is a worldwide joint disease caused by abnormal chondrocytic metabolism. However, traditional therapeutic methods aimed at anti-inflammation for early-stage disease are palliative. In the present study, we demonstrated that cepharanthine (CEP), extracted from the plant *Stephania cepharantha*, exerted protective medicinal efficacy on osteoarthritis for the first time. In our *in vitro* study, CEP suppressed the elevated expression of matrix metalloproteinases (MMPs), a disintegrin and metalloproteinase with thrombospondin motifs 5 (ADAMTS5) and inducible nitric oxide synthase (iNOS) stimulated by IL-1β or TNF-α by inhibiting the activation of MAPK and NF-κB signaling pathways, and upregulated the protein expression of aggrecan, collagen II, and Sox9. Also, CEP could reverse the reduced level of cellular autophagy in IL-1β or TNF-α–induced chondrocytes, indicating that the protective effect of CEP on osteoarthritis was achieved by restoring MAPK/NF-κB-mediated autophagy. Furthermore, in a murine OA model, CEP mitigated cartilage degradation and prevented osteoarthritis in the CEP-treated groups versus the OA group. Hence, our results revealed the therapeutic prospect of CEP for anti-osteoarthritic treatment.

## Introduction

Osteoarthritis (OA) is a multifactorial degenerative disorder that occurs predominantly in elderly individuals and postmenopausal women and is characterized by gradual articular cartilage deterioration, subchondral bone sclerosis, and osteophyte formation (Felson and Zhang, 1998; Glyn-Jones et al., 2015). Nowadays, there are approximately 10% of males and 18% of females over the age of 60 worldwide suffering from OA, and in advanced countries, the consequential cost is between 10% and 25% of the gross domestic product (Glyn-Jones et al., 2015). Also, with the aging population, the number of elderly patients and the demand for joint replacement will expand dramatically in the near future, which signifies a large socioeconomic burden (Losina et al., 2013; Chen et al., 2021).

The treatment for osteoarthritis includes pain management for early-stage and joint arthroplasty for end-stage disease (Glyn-Jones et al., 2015). For the inchoate stage of OA, non-steroidal anti-inflammatory drugs (NSAIDs) are regarded as the most efficacious therapies, but a range of corresponding side effects may occur during the treatment process, involving gastrointestinal, hepatic, renal, or cardiovascular adverse events (Bariguian Revel et al., 2020). In addition, patients with advanced-stage OA need joint replacement, but recent studies have shown that about 82% of total knee replacements (TKRs) can only last for 25 years and need joint replacement revision, which is accompanied with extra risks (GBD 2016 DALYs and HALE Collaborators, 2017; Evans et al., 2019). Thus, it is imperative to find a secure and effective drug to prevent the progression of OA.

It is reported that chronic low-grade inflammation gives rise to OA occurrence and many cytokines and chemokines are involved (Liu-Bryan and Terkeltaub, 2015). Pro-inflammatory mediators, particularly interleukin-1 beta (IL-1β) and tumor necrosis factor alpha (TNF-α), play critical roles (Yasuda, 2011). Also, these catabolic factors stimulate chondrocytes to discharge MMPs and ADAMTS by activating a train of pathways, such as the mitogen-activated protein kinase (MAPK) and NF-κB signaling, and further break extracellular matrix (ECM) proteins down, leading to cartilage degradation (Moos et al., 1999; Mengshol et al., 2000; Yang et al., 2017). Thus, targeting the inflammation-associated factors and signaling pathway–related molecules might bring about alternative and innovative therapies.

There are many signaling pathways involved in the autophagic process, and the MAPK and NF-κB pathways have been reported as upstream regulators of autophagy (Nopparat et al., 2017; He et al., 2018; Chen et al., 2020; Duan et al., 2020). Autophagy is a highly conserved degradation system that plays an important role in maintaining the homeostasis of anabolism and catabolism in cells (Mizushima, 2007). Under physiological or pathological conditions, it can remove cytoplasmic long-lived or damaged organelles and proteins to lysosomes for degradation (Nakamura and Yoshimori, 2017). Many studies have reported that autophagy mediates the steady state of articular cartilage (Caramés et al., 2012; Li et al., 2016; Duan et al., 2020). However, the autophagic activity of chondrocytes would be impaired in the process of OA (Feng et al., 2020; You et al., 2021), and the activation of autophagy can significantly slow down OA development (Qin et al., 2017). Hence, strategies to activate autophagy through pharmacological intervention have been used to prevent cartilage degradation, providing a direction for OA treatment (You et al., 2015; Saitoh and Akira, 2016).

Cepharanthine (CEP) is a natural plant extract from *Stephania cepharantha* Hayata and has various pharmacological properties, consisting of anti-inflammation, anti-virus, anti-oxidants, anti-parasites, and immunomodulation (Okamoto et al., 2001; Furusawa and Wu, 2007; Zhou et al., 2012; Paudel et al., 2016). These effects have led to its use in the treatment of malaria, alopecia areata, leukemia, thrombocytopenic purpura, and other diseases (Ohta and Morita, 1990; Morita et al., 2002; Rogosnitzky and Danks, 2011; Tabata et al., 2012). In addition, several research studies have shown that CEP could promote autophagy to exert its antiviral effects (Liu et al., 2021). However, the regulation of CEP on chondrocyte metabolism and autophagy remains elusive in OA. Here, we evaluated the effects of CEP on the variation of inflammatory responses, anabolism, and autophagy in osteoarthritic chondrocytes and elucidate its underlying mechanism.

## Materials and Methods

### Chemicals and Reagents

Cepharanthine was bought from Aladdin Chemistry Co., Ltd (C102706-5q), solubilized in DMSO, and stored at −20°C for later use in experiments. DMEM/F12 medium, FBS, pancreatic enzyme, and streptomycin/penicillin were acquired from Gibco (NY, United States). Collagenase II and DMSO were bought from Sigma-Aldrich (Merck KGaA, MO, United States). 17*β*-oestradiol (E2) was obtained from Selleck (Shanghai, China). Recombinant IL-1β and TNF-α were obtained from R&D Systems (Abingdon, United Kingdom). The radio-immuno precipitation assay (RIPA) lysis buffer and the bicinchoninic acid (BCA) assay kit were purchased from Hangzhou Fude Biological Technology Co., Ltd (Hangzhou, China). Also, 3-methyladenine (3-MA) and anisomycin were obtained from MedChemExpress (Monmouth Junction, NJ).

### Extraction, Culture, and Treatment of Primary Mice Chondrocytes

Primary chondrocytes were extracted from Days 1–3 newborn mice on the basis of the previous protocol (Gosset et al., 2008). Briefly, removal of skin and soft tissues from the hindquarters of the suckling mice was performed at first. Tibial plateau and femoral condyles were cut and collected in sterile environments. After washing with PBS, the harvested tissues were digested using 0.25% pancreatic enzyme for half an hour at 37°C to remove the soft tissue. Then cartilage pieces were retrieved and digested for a further 4 h incubating with digestion buffer (DMEM/F12 containing 0.2% collagenase II) at 37°C. The chondrocytes were harvested from centrifuged cells and subsequently seeded into Petri dishes with fresh medium (DMEM/F12 containing 10% fetal bovine serum and penicillin/streptomycin) in the constant temperature incubator until confluent. Only the first- and second-passage chondrocytes were utilized in experiments. The chondrocytes were removed and collected by trypsinization, pretreated with varying doses of CEP for 2 h, and then co-incubated with or without IL-1β (10 ng/ml) or TNF-α (10 ng/ml) for a further 24 h or 30 min.

### CCK-8 Assay

To examine the toxicity of CEP on chondrocyte viability, the CCK-8 assay (Boster Biological Technology Co., Ltd., Pleasanton, CA, United States) was administered under the manufacturer’s protocol. The second-passage chondrocytes were cultured in 96-well plates (1.5*10^4^ per well) for 24 h. Then, the cells were incubated with DMEM/F12 medium containing different concentrations of CEP (0, 0.125, 0.25, 0.5, 1, 1.25, 1.5, 1.75, 2, 4, and 8 μM) for 24, 48 h, or 72 h; the cells were subsequently exposed to 10 ng/ml IL-1β or TNF-α with or without CEP (1, 1.5, and 2 μM) for 24 h. Afterward, the medium was displaced with DMEM/F12 supplemented with 10% CCK-8 reagent, and the chondrocytes were cultured for another 3 h at 37°C. Finally, the optical density (OD) was determined at 450 nm with an ultraviolet spectrophotometer.

### RNA Isolation and Reverse Transcription-qPCR

Total RNA of chondrocytes was isolated utilizing TRIzol reagent (Invitrogen, CA, United States) and reverse-transcribed to synthesize cDNA using the PrimeScript™ RT Master Mix Kit (Takara Biotechnology Co., Ltd) after quantified. Real-time PCR was conducted using SYBR^®^ Premix Ex Taq™ II (Takara Biotechnology Co., Ltd) on an Applied Biosystems StepOnePlus™ (Applied Biosystems, United States) according to the manufacturer’s protocol. Prior to amplification, each 10-μl sample was prepared, including 5 μl of SYBR^®^ Green, 1 μl of cDNA, 0.4 μl of each primer, and 3.2 μl ddH_2_O. The sequences of all primers designed on the basis of established GenBank sequences are listed in Supplementary Table S1, and GAPDH was used as the endogenous control. The mRNA expression data of those genes were analyzed using the 2^−ΔΔCt^ formula.

### Protein Isolation and Western Blot

The whole protein of chondrocytes was isolated using 250 μl RIPA buffer (containing 0.1% phosphorylated proteinase inhibitor) for 60 min. The supernatants were collected, and the proteins were quantified by a BCA assay kit. Equal volumes of protein of each sample were separated by SDS-PAGE with a 10% gel and electrotransferred onto polyvinylidene difluoride membranes (Millipore, Bedford, MA, United States). After non-specifically blocking with 10% milk in TBST, the membranes were incubated with the specific primary antibodies against MMP13 (rabbit, cat. no. #18165-1-AP; Proteintech), MMP3 (rabbit, cat. no. #ab52915; Abcam), MMP9 (rabbit, cat. no. #ab38898; Abcam), iNOS (rabbit, cat. no. #ab3523; Abcam), Aggrecan (mouse, cat. no. #ab3778; Abcam), Collagen II (rabbit, cat. no. #ab34712; Abcam), Sox9 (rabbit, cat. no. #ab5535; Abcam); Atg7 (rabbit, cat. no. #8558; Cell Signaling Technology, Inc.), Beclin-1 (rabbit, cat. no. #3495; Cell Signaling Technology, Inc.), LC3 (rabbit, cat. no. #2775; Cell Signaling Technology, Inc.); NF-κB p65 (rabbit, cat. no. #4764S; Cell Signaling Technology, Inc.), pp65 (Ser536) (rabbit, cat. no. #3031; Cell Signaling Technology, Inc.); MAPK p38 (rabbit, cat. no. #9212; Cell Signaling Technology, Inc.), pp38 (rabbit, cat. no. #4511; Cell Signaling Technology, Inc.), Jnk (rabbit, cat. no. #9258; Cell Signaling Technology, Inc.), p-Jnk (rabbit, cat. no. #4668; Cell Signaling Technology, Inc.), Erk (rabbit, cat. no. #4695; Cell Signaling Technology, Inc.), p-Erk (rabbit, cat. no. #9101; Cell Signaling Technology, Inc.), and *β*-actin (mouse, cat. no. #ab8226; Abcam) at 4°C overnight. Also, β-actin was utilized as the endogenous control. The next day, the membranes were washed three times and probed with the corresponding secondary antibody at room temperature for 1 h. After further three washes, the protein bands were viewed through the Bio-Rad ChemiDoc system (Bio-Rad Laboratories, Inc.) using an ECL kit (Millipore, Billerica, MA, United States).

### Animal Experiments

All experiments involving animals were approved by the Ethics Committee of The Second Affiliated Hospital, School of Medicine, Zhejiang University (Hangzhou, China). A total of 24 8-week-old male C57BL/6 mice were used for our *in vivo* experiment and divided into four groups, namely, Sham, OA, low-dose CEP- and high-dose CEP-treated groups (*n* = 6 in each group). Mice were provided with standard mice chow and water routinely and were housed in a clean vivarium (specific pathogen free) at room temperature with 12 h day/night cycle.

Experimentally, the destabilization of the medial meniscus (DMM) surgery was operated on mice to induce osteoarthritis as previously described (Glasson et al., 2007). Concisely, pentobarbital was injected into the peritoneal cavity, and the mice were anesthetized. Then, the knee joint was exposed and opened with the medial parapatellar incision. The medial meniscotibial ligament (MMTL) was transected in the OA- and CEP-treated groups without any ligament or cartilage injuries, while the sham group received a similar incision without MMTL transection. One week after surgery, 5 and 15 mg/kg CEP were injected into the peritoneal cavity of mice in the low-dose CEP- and high-dose CEP-treated groups, respectively, once a week. Meanwhile, the mice in the Sham and OA group were injected with physiological saline. After 12 weeks, the mice were euthanized, and the knee joint was fixed in tissue fixation solution. Then, the specimens were decalcified for 3 weeks with 10% EDTA and embedded into paraffin.

### Histological Analysis

The knee joint specimens were dissected into 5-μm sections from the paraffin blocks using a microtome. After deparaffinization with xylene and gradient hydration, the safranin O/Fast Green and hematoxylin and eosin staining (H&E staining) were performed for the evaluation of articular cartilage destruction according to the instructions. Histological assessment was carried out by three blinded individuals *via* the OARSI scoring system based on the structural and morphological changes of the cartilage, the loss of safranin, and the degree of joint erosion.

### Immunofluorescence and Immunohistochemical Analysis

Immunofluorescence staining was performed on the sagittal sections of the murine knee joint to estimate the severity of OA. The incubation of primary antibody against MMP13 (rabbit, cat. no. #18165-1-AP; Proteintech) and collagen II (rabbit, cat. no. #ab34712; Abcam) on the slices was administered after antigen retrieval and blocking. Then, the sections were probed with fluorescein isothiocyanate–conjugated secondary antibodies (Alexa Fluor 555-labeled Donkey Anti-Rabbit IgG (H + L) and Alexa Fluor 488-labeled Donkey Anti-Rabbit IgG (H + L), Beyotime Institute of Biotechnology). The nuclei were counterstained with DAPI (Sigma, D9542). The images were viewed using a Nikon A1 confocal microscope and analyzed using ImageJ software.

For immunohistochemistry, the knee slices were prepared as described in the histological analysis and subsequently subjected to antibodies against MMP3 (rabbit, cat. no. #ab52915; Abcam) and ADAMTS5 (rabbit, cat. no. #ab41037; Abcam). Percentages of positive MMP3 and ADAMTS5 chondrocytes were determined by counting the number of immunostained cells and dividing by the total number of chondrocytes visualized by a hematoxylin counterstain.

### Statistical Analysis

All experiments were repeated in triplicate independently, and the statistics were analyzed using Prism 8.00, GraphPad Software. All data were expressed as the mean ± SD. One-way ANOVA followed by Tukey’s post hoc test was performed to determine the statistically significant difference between different experimental groups. Also, the value of *p* < 0.05 was set as the threshold of statistical significance.

## Results

### The Effect of CEP on Chondrocyte Viability

**FIGURE 1:**
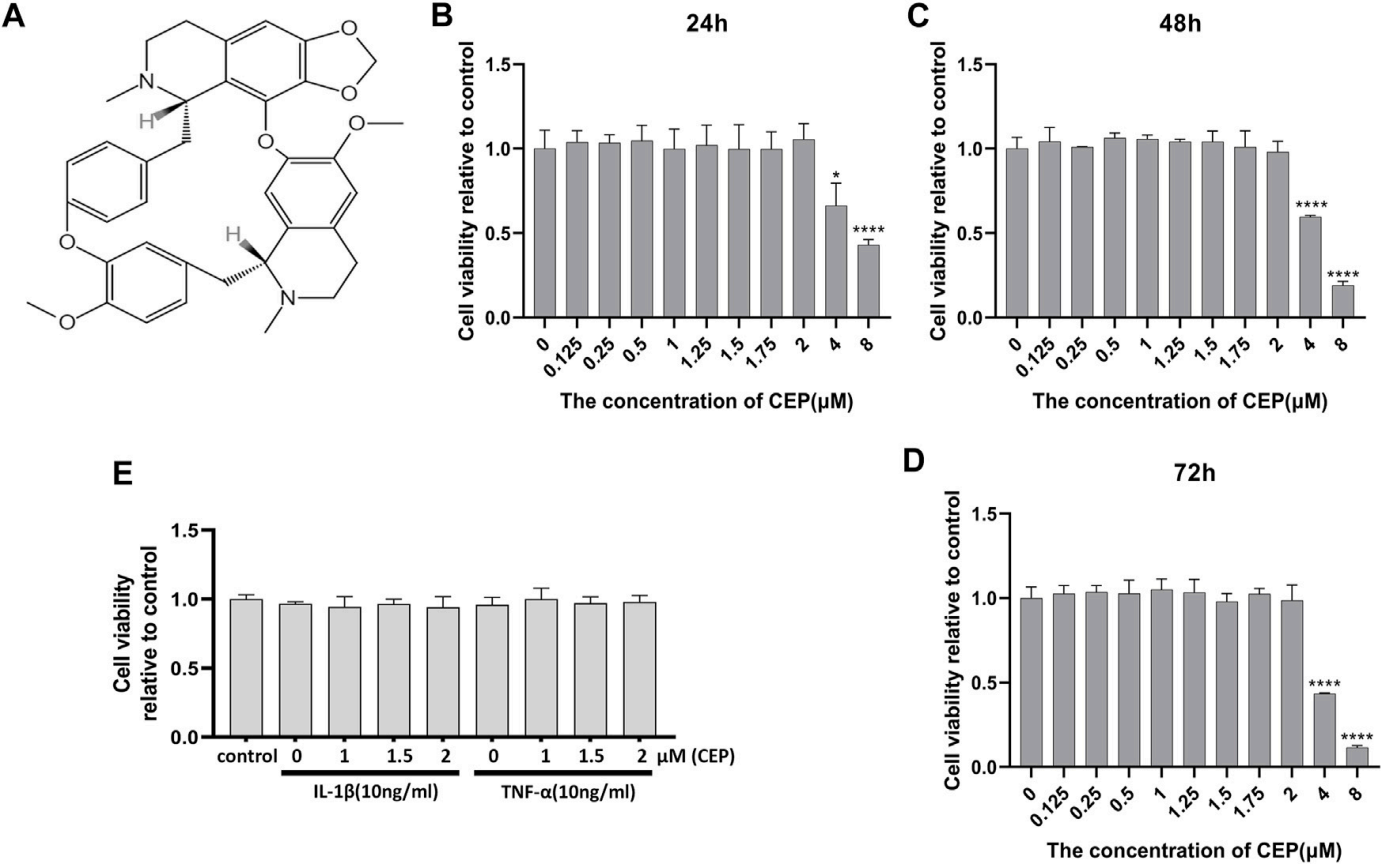
Effects of CEP on chondrocyte viability. **(A)** Molecular structure of CEP. **(B,C,D)** Cells were treated with the indicated concentrations of CEP for 24, 48, and 72 h. Mouse chondrocyte viability was evaluated by CCK-8 assay. **(E)** Chondrocytes were exposed to 10 ng/ml IL-1β or TNF-α with or without CEP (1, 1.5, and 2 μM), and cell viability was determined by CCK-8 assay. All experiments were repeated independently three times. Values are expressed as mean ± SD; *p < 0.05 and ****p < 0.0001 vs. the control group.

The molecular structural formula of CEP is shown in Figure 1A. In the first place, the cytotoxicity of CEP on chondrocyte viability was determined by the CCK-8 assay. As shown in Figures 1B–D, ≤2 μM CEP had no obvious toxicity to mouse chondrocytes at 24, 48, and 72 h. Thus, the concentrations of 1, 1.5, and 2 μM were selected to use for further *in vitro* experiments.

### CEP Suppressed the Expression Levels of Matrix-Degrading Genes and Inflammation-Induced Cartilage Degradation

**FIGURE 2:**
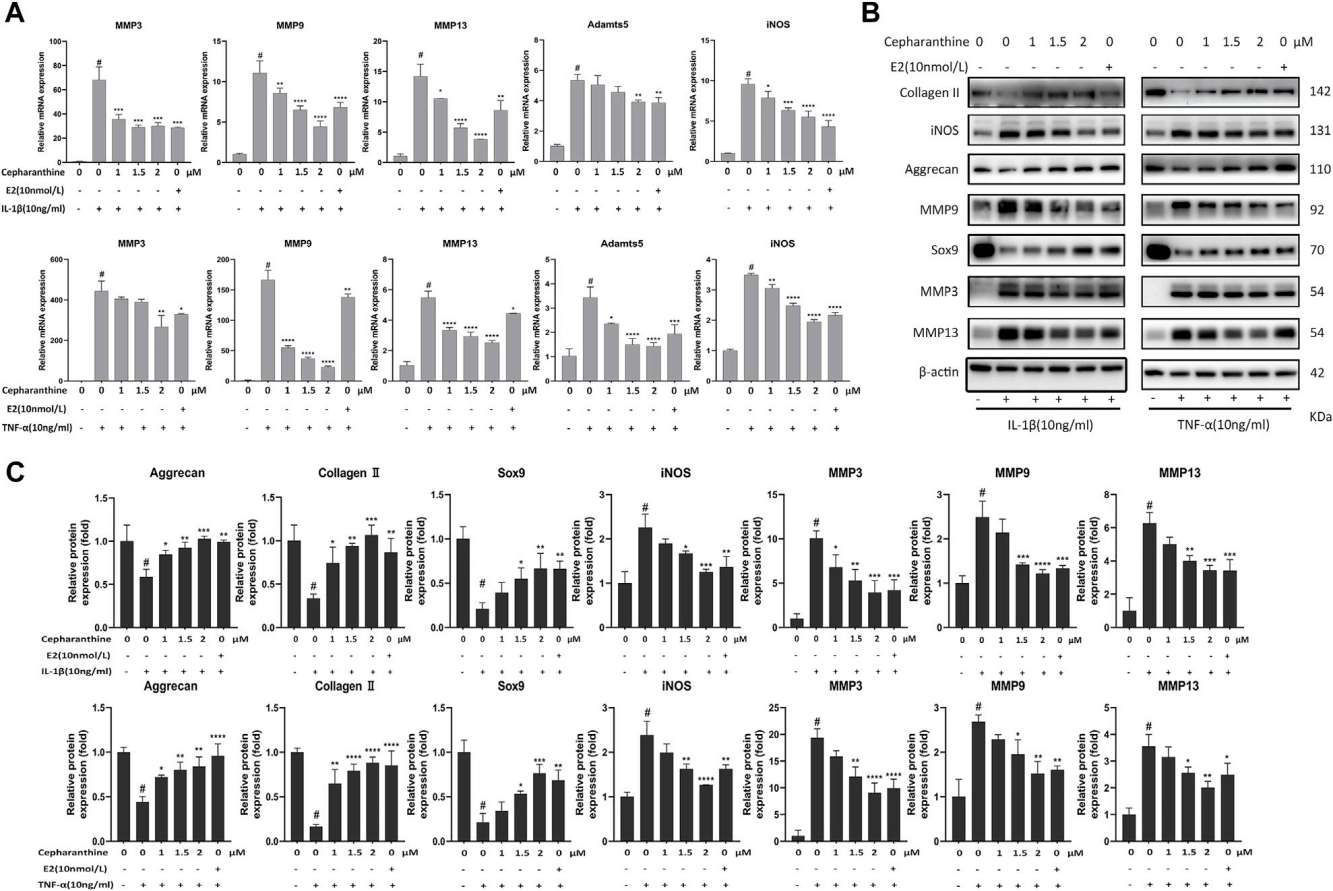
CEP suppressed the expression levels of matrix-degrading genes and inflammation-induced cartilage degradation. CEP-pretreated chondrocytes were incubated with IL-1β or TNF-α at 10 ng/ml for 24 h. **(A)** Gene expression analysis of MMP3, MMP9, MMP13, Adamts5, and iNOS in mouse chondrocytes treated with IL-1β or TNF-α. **(B,C)** Representative Western blots and quantification data of collagen II, iNOS, aggrecan, MMP9, Sox9, MMP3, and MMP13 in mouse chondrocytes after treatment with IL-1β or TNF-α, respectively. Values are expressed as mean ± SD, *n* = 3; #*p* < 0.05 vs. control group, *p < 0.05, **p < 0.01, ***p < 0.001, and ****p < 0.0001 vs. model group.

In order to investigate the protective effect of CEP against cartilage inflammation, we performed a series of *in vitro* experiments. As IL-1β and TNF-α are common joint inflammation elements, we administered these two factors to induce chondrocytic inflammation. Also, CCK-8 results showed that 10 ng/ml IL-1β or TNF-α with or without CEP (1, 1.5, and 2 μM) also had no effect on cell viability (Figure 1E). It is reported that 17β-oestradiol (E2) can abate IL-1β–induced phosphatidyl glycerol degradation and nitric oxide synthase expression in chondrocytes (Richette et al., 2004; Richette et al., 2007) and protect chondrocytes against oxygen radical–induced damage (Claassen et al., 2005). So, we used it here as a positive control to observe the anti-inflammatory effect of CEP. RT-qPCR and Western blot were utilized to test the expression of MMP3, MMP9, MMP13, Adamts5, iNOS, aggrecan, collagen II, and Sox9. The RT-qPCR results indicated that CEP could decrease the upregulation of matrix-degrading enzymes (MMP3, MMP9, MMP13, Adamts5, and iNOS) induced by IL-1β or TNF-α (Figure 2A). Also, the results of Western blot also showed that the increased protein expression levels of MMPs and iNOS were reversed in CEP groups in a dose-dependent manner (Figures 2B,C). In the meantime, CEP inhibited the downregulation of extracellular matrix components (collagen II and aggrecan) and Sox9 stimulated by IL-1β or TNF-α at the protein level, especially at 2 μM (Figures 2B,C). Hence, these data revealed that CEP could protect mouse chondrocytes by suppressing the expression of inflammatory and matrix-degrading genes and upregulating cartilage-specific gene expression.

### CEP Inhibited the Activation of MAPK and NF-κB Signaling Pathways Induced by IL-1β or TNF-α in Chondrocytes

**FIGURE 3:**
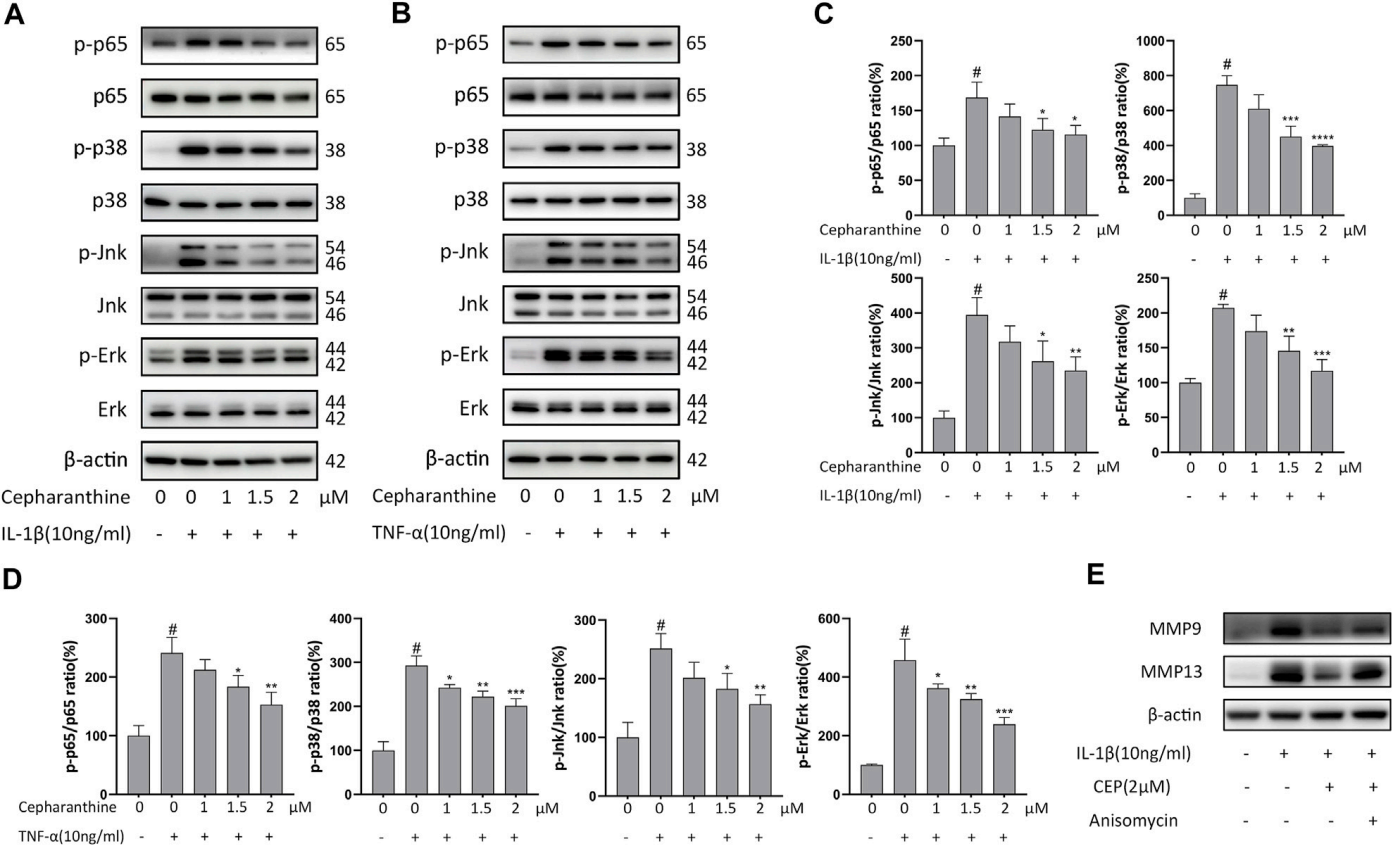
CEP inhibited the activation of MAPK and NF-κB signaling pathways induced by IL-1β or TNF-α in chondrocytes. Chondrocytes were pretreated with various concentrations of CEP for 2 h and subsequently stimulated with 10 ng/ml IL-1β or TNF-α for 30 min **(A,B)** Western blot analysis of p65, p-p65, p-38, p-p38, Erk, p-Erk, Jnk, and p-Jnk after IL-1β or TNF-α treatment. **(C,D)** Quantitative analysis relevant to p-p65/p65, p-p38/p38, p-Jnk/Jnk, and p-Erk/Erk after treatment with IL-1β or TNF-α, respectively. **(E)** Western blotting of MMP9 and MMP13 in chondrocytes pretreated with or without anisomycin (10 μM) before treatment with CEP (2 μM) or IL-1β. Values are expressed as mean ± SD, *n* = 3; #*p* < 0.05 vs. control group, *p < 0.05, **p < 0.01, ***p < 0.001, and ****p < 0.0001 vs. model group.

To determine the underlying mechanism by which CEP protected the cartilage against degradation, we next investigated the MAPK and NF-κB signaling pathways. The Western blot results manifested that IL-1β or TNF-α activated MAPK and NF-κB signaling pathways and increased the levels of phosphor-p65, phosphor-p38, phosphor-Erk, and phosphor-Jnk. But, CEP abated the enhanced phosphorylation levels of p-p65, p-p38, p-Erk, and p-Jnk significantly (Figures 3A,B). Moreover, CEP remarkably inhibited the elevation of the p-p65/p65, p-p38/p38, p-Jnk/Jnk, and p-Erk/Erk ratios stimulated by IL-1β or TNF-α (Figures 3C,D). Then, we found that anisomycin, a JNK agonist, eradicated the anti-inflammatory effect of CEP partly (Figure 3E). Thus, these results suggested that CEP could suppress the activation of MAPK and NF-κB signaling pathways induced by IL-1β or TNF-α in chondrocytes.

### CEP Attenuated Downregulation of Autophagy in Chondrocytes

**FIGURE 4:**
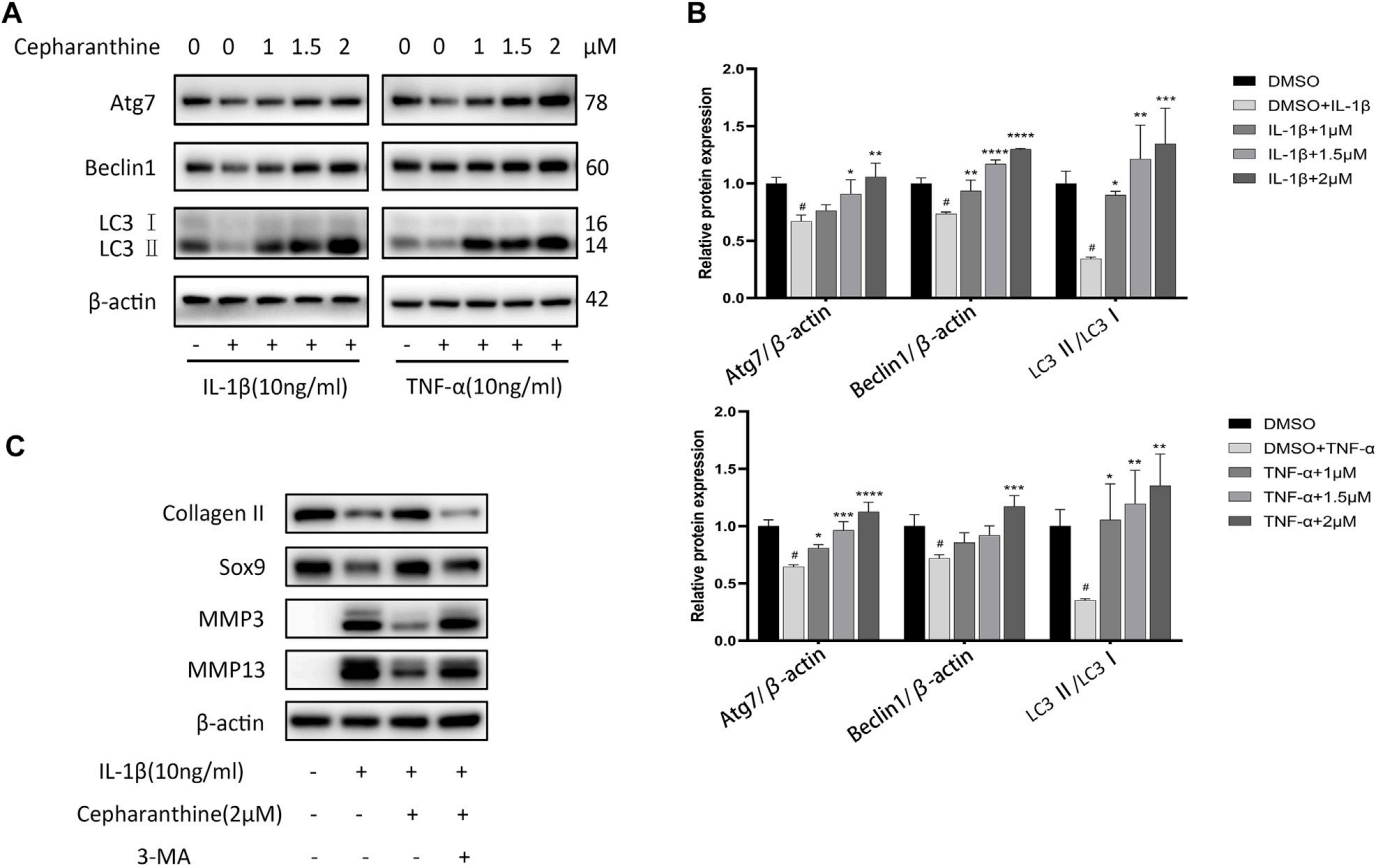
CEP attenuated downregulation of autophagy in chondrocytes. **(A,B)** Western blot results and quantitative analysis showed the levels of Atg7, beclin1, and LC3 II/LC3 I ratio in chondrocytes stimulated with IL-1β or TNF-α for 24 h after CEP pretreatment. **(C)** Western blotting of collagen II, Sox9, MMP3, and MMP13 in chondrocytes pretreated with or without 3-MA (5 mM) before treatment with CEP (2 μM) or IL-1β. Values are expressed as mean ± SD, *n* = 3; #*p* < 0.05 vs. control group, *p < 0.05, **p < 0.01, ***p < 0.001, and ****p < 0.0001 vs. model group.

Autophagy, as a protective mechanism in cells, has been proven to prevent chondrocytes from degeneration and mitigate the occurrence and progression of OA (Caramés et al., 2012). Also, several research studies have shown that NF-κB could mediate autophagy *via* suppressing Beclin1, the initiator of autophagy, and the MAPK pathway could also inhibit autophagy (Nopparat et al., 2017; Duan et al., 2020). To determine whether the protective role of CEP on OA is associated with autophagy, the cells were incubated with IL-1β or TNF-α after pretreating DMSO or CEP for 2 h, and autophagy-related proteins were detected, such as Atg7, beclin1, and LC3II/LC3I ratio. As indicated in Figures 4A,B, IL-1β or TNF-α treatment decreased the expression of beclin-1 and ATG7 and reduced the ratio of LC3II/LC3I, implying that the level of autophagy is inhibited. Nevertheless, CEP reversed the manifestation notably. LC3II/I is regarded as the mark of autophagy, and CEP greatly promoted the LC3II/I ratio at 2μM, even higher than the normal group (Figure 4B), suggesting that CEP could act as an autophagy promoter to counteract OA. Also, the autophagy inhibitor 3-MA was utilized in our experiment. The cells were pretreated with 3-MA (5 mM) for 2 h and then stimulated with IL-1β for 24 h with or without CEP (2 μM) pretreatment. As shown in Figure 4C, CEP decreased the expression of MMP3 and MMP13 and increased the expression of collagen II and Sox9. But, 3-MA reversed the effect of CEP. Therefore, CEP protection of chondrocytes against inflammation and ECM degradation is achieved by activating MAPK-/NF-κB–mediated autophagy.

### CEP Prevented Osteoarthritis *in vivo*

**FIGURE 5:**
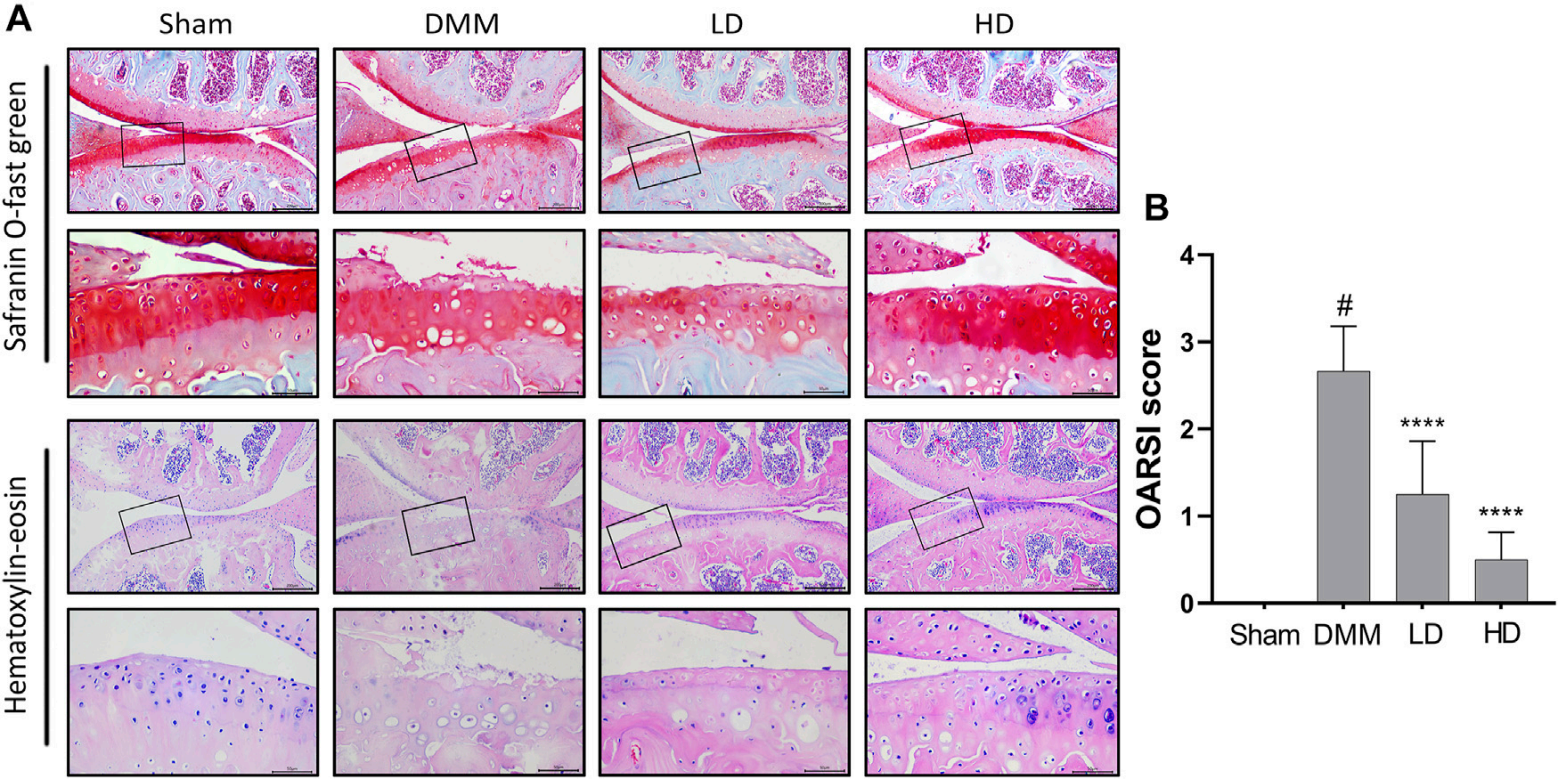
Effect of CEP on cartilage degradation in DMM-induced OA pathogenesis. **(A)** Representative images of safranin O staining and H&E staining of knee joints in four groups (*n* = 6 per group). Scale bar = 200 µm. The framed area in each picture is shown below at a higher magnification. **(B)** Quantitation of OA severity by the OARSI score for mice in Figure 5A. LD and HD represent the dose of 5 and 15 mg/kg CEP, respectively. Values are expressed as mean ± SD, n = 6. #*p* < 0.05 vs. sham group, and ****p < 0.001 vs. OA group.

To determine the effects of CEP on articular cartilage maintenance *in vivo*, we used a murine model of DMM-induced osteoarthritis and treated mice with either vehicle or different concentrations of CEP. H&E staining indicated that different concentrations of CEP have no toxicity to the liver and kidney (Supplementary Figures S1A, B). As illustrated in Figure 5A, the OA group exhibited OA characteristics such that the integrity of articular cartilage was significantly damaged compared with the sham group. However, a protective effect was observed in the groups treated with CEP, especially in the high-dose group. Also, the anti-osteoarthritic characteristics of CEP were verified by using OARSI score analysis (Figure 5B).

**FIGURE 6:**
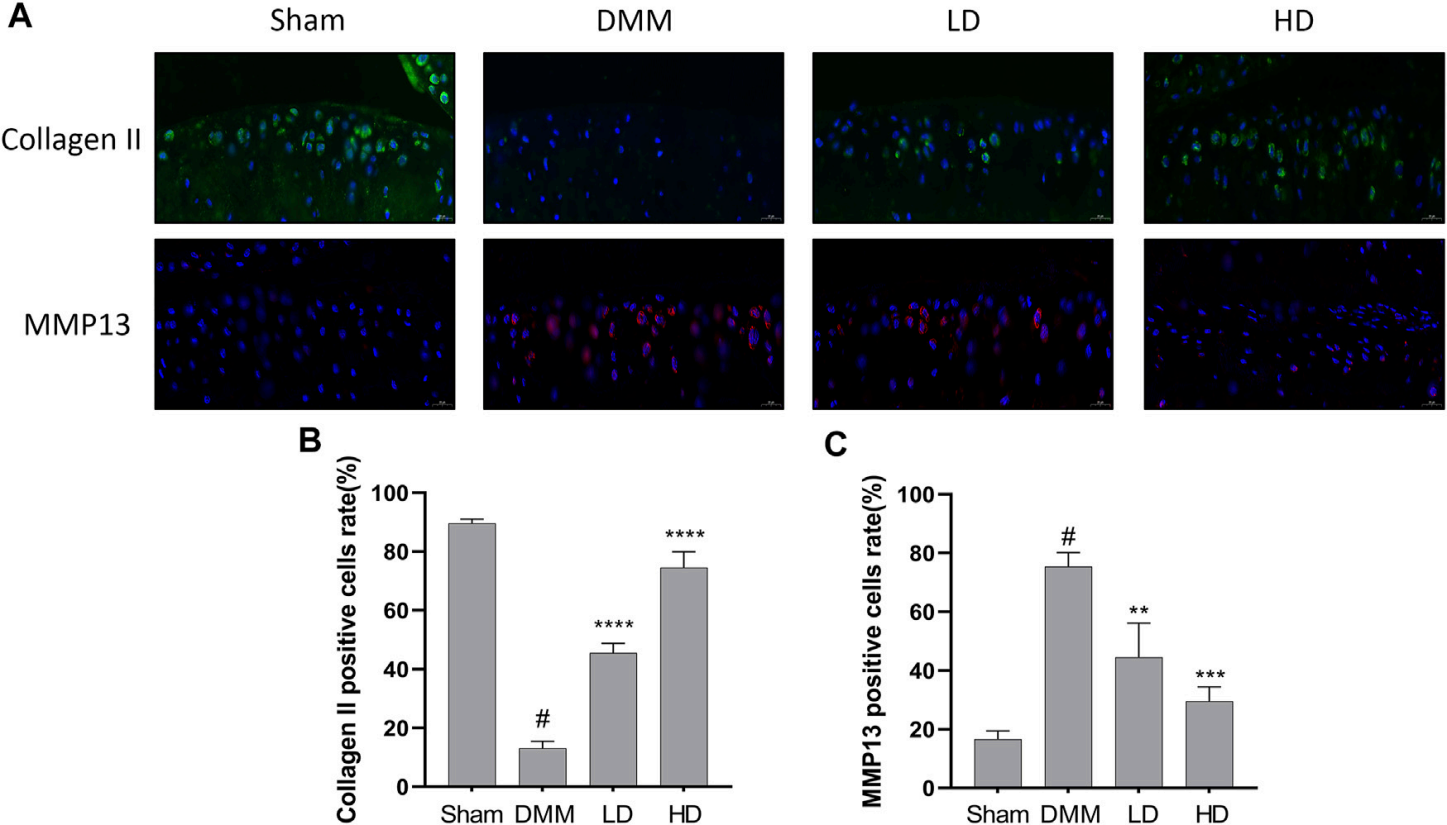
Protective characteristics of CEP in a murine OA model. Immunofluorescence for antibody against collagen II and MMP13 **(A)**, and quantitative analysis **(B,C)**. Green, collagen II; Red, MMP13; Blue, DAPI. Scale bar = 20 μm. LD and HD represent the dose of 5 and 15 mg/kg CEP, respectively. Values are expressed as mean ± SD, *n* = 6. #*p* < 0.05 vs. sham group, **p < 0.01, ***p < 0.001, and ****p < 0.0001 vs. OA group.

**FIGURE 7:**
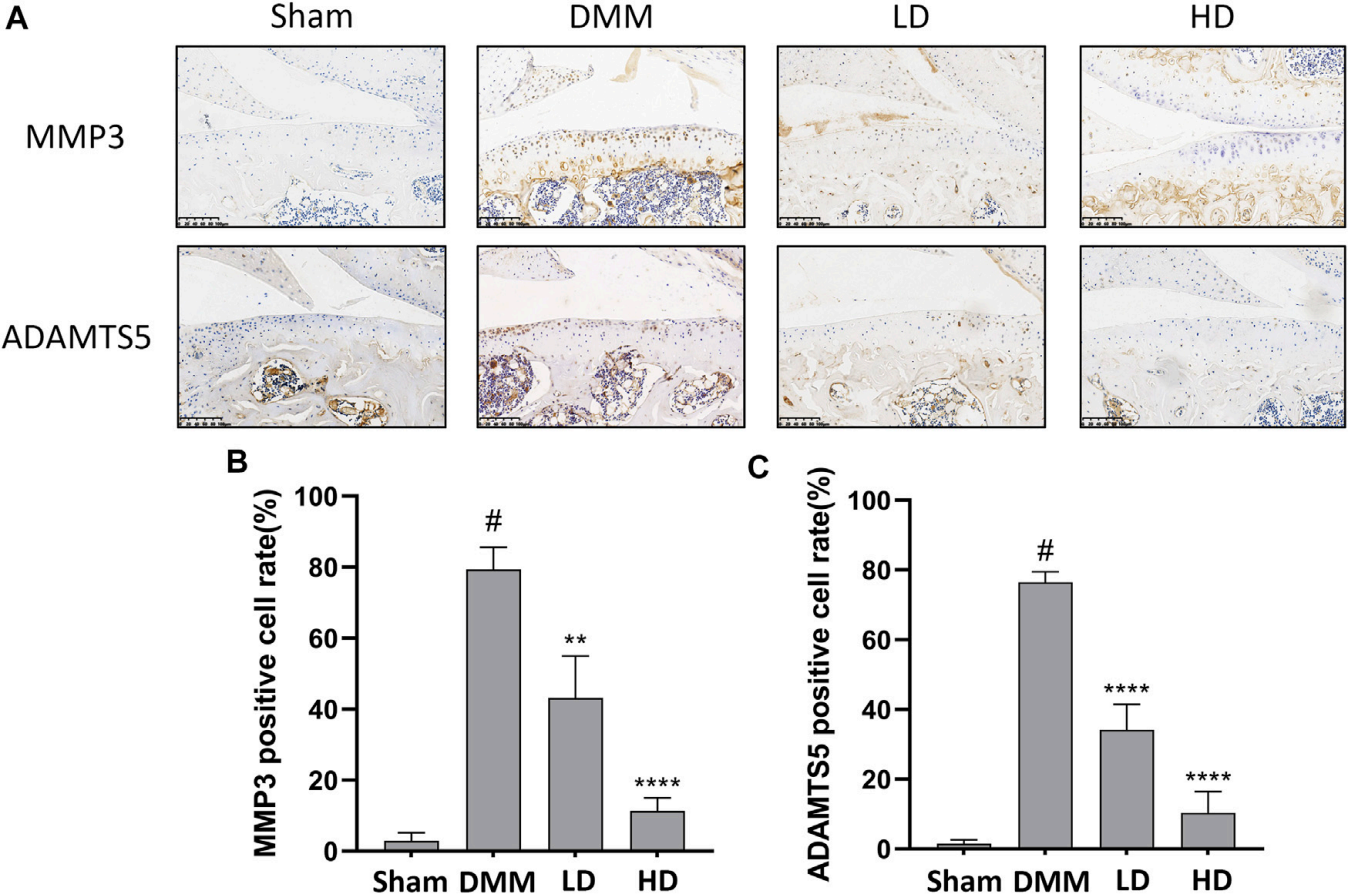
CEP ameliorated cartilage inflammation *in vivo*. Immunohistochemistry **(A)** and quantitative analysis **(B,C)** for antibody against MMP3 and ADAMTS5. Scale bar = 100 μm. LD and HD represent the dose of 5 and 15 mg/kg CEP, respectively. Values are expressed as mean ± SD, *n* = 6. #*p* < 0.05 vs. sham group, **p < 0.01, and ****p < 0.0001 vs. OA group.

To further confirm the preventive effects of CEP on DMM-induced osteoarthritis, we examined the expression of some OA-related proteins in tissues by immunofluorescence and immunohistochemistry. The results showed that collagen II expression remarkably increased in CEP-treated groups compared to the OA group, while the expression of MMP13, MMP3, and ADAMTS5 decreased markedly (Figures 6, 7). Collectively, these data demonstrated that CEP has a protective effect on DMM-induced osteoarthritis *in vivo*.

## Discussion

Chondrocytes, as the unique cell type in cartilage, together with their synthesized and secreted extracellular matrix, make up the articular cartilage (Charlier et al., 2019; Rim et al., 2020). A fine balance between anabolism and catabolism can maintain the homeostatic state of chondrocyte metabolism. However, under stress conditions, including inflammation, metabolic syndrome, mechanical overload, and aging, the chondrocyte would switch to a metabolically imbalanced state, resulting in the occurrence of OA finally (Zheng et al., 2021). A variety of drugs are applied to treat osteoarthritis, but traditional pharmacotherapy is described as reactive and palliative in the early stage of OA and has potential, adverse side effects (Hunter et al., 2014). In this research, we attested that CEP showed its protective effects on osteoarthritis by inhibiting catabolism (MMPs and ADAMTS) and enhancing anabolism (aggrecan and collagen II) *in vitro* and *in vivo*. Moreover, we demonstrated that CEP ameliorated osteoarthritis by downregulating the MAPK/NF-κB signaling pathways and restoring MAPK/NF-κB–mediated autophagy. As a result, our findings suggested that CEP might be a promising compound for the treatment of OA.

There are several natural products showing potential in the prevention of osteoarthritis, and most of them are limited to preclinical research studies apart from curcumin and resveratrol (Chin, 2016; Ran et al., 2018; Moqbel et al., 2020; Tian et al., 2021). Among them, curcumin exhibits powerful chondroprotective effects by restoring the expression of chondrocyte-specific proteins such as aggrecan and collagen II significantly based on its anti-inflammatory and antiviral properties and has been used clinically to improve pain in OA patients (Tian et al., 2021; Wang et al., 2021). However, the low bioavailability and poor stability of curcumin in the body limit its long-term use for OA treatment (Panahi et al., 2014). Also, the adverse effects of most natural compounds in the long-term application are still unknown. CEP has been clinically used for diverse diseases in Japan for decades, and no serious side effects have been reported until now, even in tumor treatment where a larger dosage has been applied (Takayanagi et al., 2002; Ita et al., 2008). Previous studies have indicated that CEP has anti-inflammatory and antioxidant pharmacological characteristics (Okamoto et al., 2001; Furusawa and Wu, 2007). Meanwhile, chronic inflammation and oxidative stress play a critical role in the pathological progression of OA (Liu-Bryan and Terkeltaub, 2015). Thus, we speculated that CEP might inhibit inflammation and ECM degradation induced by IL-1β or TNF-α *in vitro*. First, we observed that it has no obvious cytotoxicity to chondrocytes when the concentration of CEP is between 0 and 2 μM. Then, we demonstrated the chondroprotective effect of CEP on anti-inflammation and extracellular matrix proteins.

The degeneration of chondrocytes is a vital part of the progression of OA, and inflammatory elements such as IL-1β and TNF-α are regarded as the main cause of chondrocyte metabolic disorders (Fukui et al., 2003). Our data also demonstrated that the stimulation of IL-1β or TNF-α caused the dysregulation of anabolism and catabolism in chondrocytes. Nevertheless, the increase of MMPs, ADAMTS, and iNOS and downregulation of matrix-synthesizing proteins are reversed by CEP, suggesting that CEP can maintain the dynamic balance of chondrocytes. Interestingly, we found that CEP did not ameliorate the decreased expression of cartilage-specific genes (aggrecan, Col2a1, and Sox9) in chondrocytes challenged with IL-1β or TNF-α at the mRNA level (Supplementary Figure S2), implying that CEP may act at the posttranscriptional and posttranslational level on these genes, which requires further studies to confirm. 17β-oestradiol (E2), as the OA-antagonized hormone, also has no effect on the decrease of cartilage-specific gene mRNA expression induced by IL-1β or TNF-α in our data (Supplementary Figure S2), which is in accordance with previous research studies (Richette et al., 2004). This may be because E2 plays its role by acting on RNA-binding proteins in the modulation of RNA processing.

Multiple signaling pathways are involved in the development of osteoarthritis, particularly the NF-κB and MAPK pathways (Saklatvala, 2007). Activation of the NF-κB pathway can give rise to chondrocyte-related inflammation responses and the production of MMPs, while the MAPK pathway also serves an important role in ECM metabolic imbalance and cartilage degradation, and the former has been considered a prospective therapy target in osteoarthritis (Roman-Blas and Jimenez, 2006; Malemud, 2017; Zhou et al., 2019). In the resting state, p65 exists in a complex with its inhibitory protein IκB in the cytoplasm. When activated by inflammatory mediators, p65 is released with phosphorylation, promptly translocates to the cell nucleus and upregulated the expression of inflammatory genes such as MMPs and ADAMTS, and further results in matrix degradation, acting synergistically with the MAPK pathway (Rigoglou and Papavassiliou, 2013). On the contrary, inhibition of NF-κB or the MAPK pathways can delay the development of osteoarthritis by suppressing the expression or activity of OA-related components (Malemud et al., 2003; Sondergaard et al., 2010). In our study, IL-1β or TNF-α clearly induced the phosphorylation of p65, p38, Erk, and Jnk, while CEP decreased the phosphorylation level and inhibited the activation of NF-κB and MAPK pathways.

Autophagy, as an intracellular self-renewal mechanism, maintains the structure and function of cells and provides a latent target for OA treatment (Mizushima, 2007; Appleton, 2018). Under pathological conditions, excessive reactive oxygen species (ROS) in chondrocytes accumulates and cannot be eliminated by impaired autophagy, resulting in the activation of MMPs and inflammation response (Duan et al., 2020). Recently, it is reported that CEP could suppress herpes simplex virus 1 by enhancing interferon-independent autophagy and counteract human cervical cancer *via* increasing autophagic influx (Law et al., 2014; Liu et al., 2021), and it has been demonstrated that inhibition of NF-κB could alleviate the reduction of autophagy and the MAPK pathway could inhibit autophagy by activating mTOR, a negative regulator in the process of autophagy (Chen et al., 2020; Duan et al., 2020). Here, our results exhibited that CEP could induce autophagy alone (Supplementary Figure S3) and increase the level of autophagy in chondrocytes obviously, marked by upregulation of Atg7, beclin1, and LC3II/LC3I, implying that CEP exerts its protective effects *via* promoting MAPK/NF-κB–mediated autophagy. Nonetheless, previous studies have also shown that CEP can be used as an autophagy inhibitor against non–small cell lung cancer (Tang et al., 2018). This might be due to the fact that CEP plays biphasic effects in different cell types. As an illustration, paclitaxel enhanced autophagy in non–small cell lung cancer, while it suppressed autophagy in breast cancer cells (Xi et al., 2011; Veldhoen et al., 2013).

Finally, in the animal experiment, the DMM group exhibited obvious cartilage erosion, while the CEP treatment group showed part of alleviation, particularly in the high-dose group. Moreover, the increased expression of collagen II and reduced expression of MMP13, MMP3, and ADAMTS5 were examined by immunofluorescence and immunohistochemistry. But, there are a few indicators to assess the protective role of CEP in our *in vivo* experiment, which requires further *in vivo* characterization and functional experiments to demonstrate this.

**FIGURE 8:**
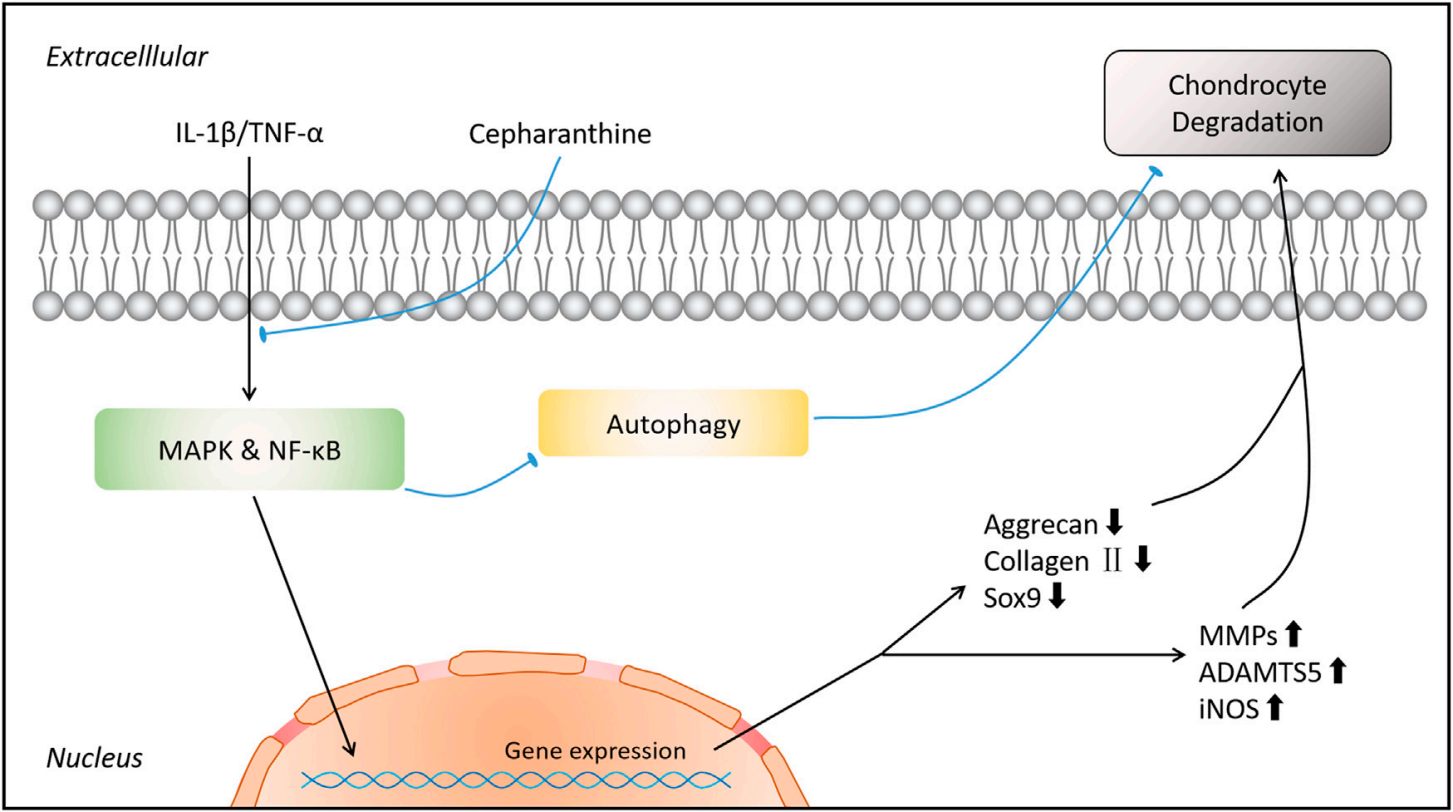
Schematic diagram indicates the potential protective effect of CEP on osteoarthritis.

In conclusion, this research revealed that CEP exerted its inhibiting actions on the expression of matrix-degrading genes and ECM destruction *via* impairing the MAPK and NF-κB signaling pathways and restoring MAPK/NF-κB-mediated autophagy *in vitro*. Moreover, we demonstrated that CEP showed a positive influence in preventing DMM-induced osteoarthritis and cartilage degradation *in vivo*. Hence, we can draw a conclusion that CEP might be an promising agent for the treatment of osteoarthritis (Figure 8).

## Data Availability

The original contributions presented in the study are included in the article/Supplementary Materials; further inquiries can be directed to the corresponding authors.
