# Supplementary material for: Cepharanthine Ameliorates Chondrocytic Inflammation and Osteoarthritis via Regulating the MAPK/NF-κB-Autophagy Pathway
Source: Front Pharmacol. 2022 Jun 21;13:854239. doi: 10.3389/fphar.2022.854239 (PMC9253373; doi:10.3389/fphar.2022.854239)
Supplement: Supplementary file 1 [file DataSheet1.pdf]

Cepharanthine ameliorates chondrocytic inflammation and osteoarthritis via regulating MAPK/NF-κB-autophagy pathway

Minjun Yao[1,2†], Caihua Zhang[3†], Lingzhi Ni[1,4†], Xiaoxiao Ji[1,2], Jianqiao Hong[1,2], Yazhou Chen[1,2], Jie Wang[1,2], Congsun Li[1,2], Jiyan Lin[1,2], Tingting Lu[5], Yihao Sheng[6], Menghao Sun[1,2], Mingmin Shi[1,2], Chenhe Zhou[1,2*] and Xunzi Cai[1,2*]

Figure S1

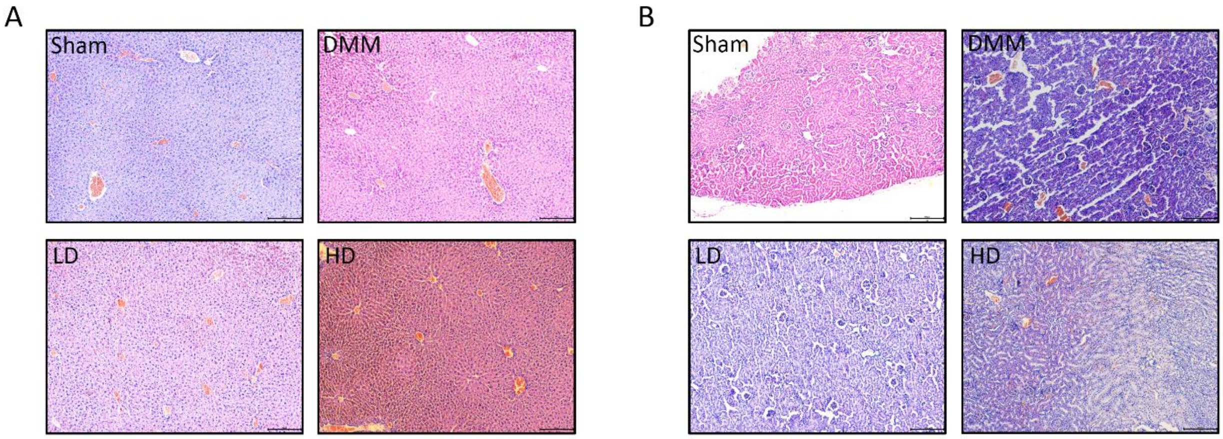

Influences of CEP on liver and kidney in a murine OA model. (A) Liver slices stained with H&E staining. (B) Kidney slices stained with H&E staining. LD and HD represent the dose of 5 and 15 mg/kg CEP, respectively. Scale bar = 500μm.

Figure S2

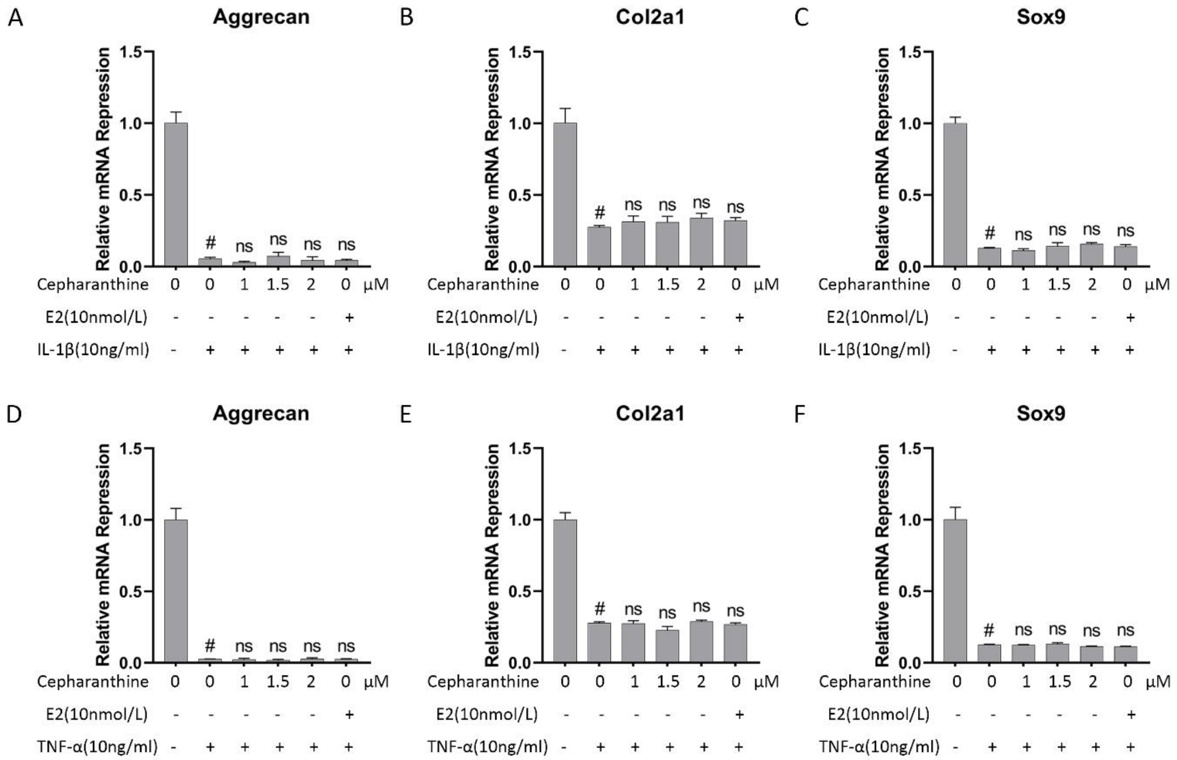

CEP did not ameliorate the downregulation of cartilage-specfic genes at the mRNA level. (A-F) Gene expression analysis of Aggrecan, Col2a1 and Sox9 in mouse chondrocytes stimulated by IL-1β or TNF-α respectively after CEP treatment. Values are expressed as mean ± SD, n = 3; #P <0.0001 vs. control group, ns.P>0.05 vs. model group.

Figure S3

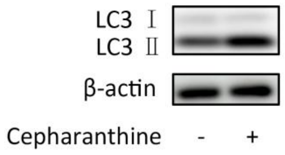

CEP could induce autophagy alone. Western blot results showed the level of LC3 II/LC3 I ratio in chondrocytes treated with or without CEP (1μM) for 24h.

Table S1

The sequences of RT-qPCR primers	
**Name**	**Sequence (5'-3')**
MMP3-F	ACATGGAGACTTTGTCCCTTTTG
MMP3-R	TTGGCTGAGTGGTAGAGTCCC
MMP9-F	GGACCCGAAGCGGACATTG
MMP9-R	CGTCGTCGAAATGGGCATCT
MMP13-F	CTTCTTCTTGTTGAGCTGGACTC
MMP13-F	CTGTGGAGGTCACTGTAGACT
Adamts5-F	GGAGCGAGGCCATTTACAAC
Adamts5-R	CGTAGACAAGGTAGCCCACTTT
COX-2-F	TGAGCAACTATTCCAAACCAGC
COX-2-R	GCACGTAGTCTTCGATCACTATC
iNOS-F	GTTCTCAGCCCAACAATACAAGA
iNOS-R	GTGGACGGGTCGATGTCAC
GAPDH-F	AGGTCGGTGTGAACGGATTTG
GAPDH-R	TGTAGACCATGTAGTTGAGGTCA
